# Analysis of the Tomato *mTERF* Gene Family and Study of the Stress Resistance Function of *SLmTERF-13*

**DOI:** 10.3390/plants12152862

**Published:** 2023-08-03

**Authors:** Ao Su, Siyu Ge, Boyan Zhou, Ziyu Wang, Liping Zhou, Ziwei Zhang, Xiaoyu Yan, Yu Wang, Dalong Li, He Zhang, Xiangyang Xu, Tingting Zhao

**Affiliations:** 1 Tomato Research Institute, College of Horticulture and Landscape Architecture, Northeast Agricultural University, Harbin 150030, China; suao1526376@163.com (A.S.); 15845192044@163.com (S.G.);; 2 Key Laboratory of Biology and Genetic Improvement of Horticultural Crops (Northeast Region), Ministry of Agriculture and Rural Affairs, Northeast Agricultural University, Harbin 150030, China

**Keywords:** tomato, *SLmTERF13*, gene family, gene silencing, stress resistance

## Abstract

Mitochondrial transcription termination factor (mTERF) is a DNA-binding protein that is encoded by nuclear genes, ultimately functions in mitochondria and can affect gene expression. By combining with mitochondrial nucleic acids, mTERF regulates the replication, transcription and translation of mitochondrial genes and plays an important role in the response of plants to abiotic stress. However, there are few studies on *mTERF* genes in tomato, which limits the in-depth study and utilization of mTERF family genes in tomato stress resistance regulation. In this study, a total of 28 *mTERF* gene family members were obtained through genome-wide mining and identification of the tomato *mTERF* gene family. Bioinformatics analysis showed that all members of the family contained environmental stress or hormone response elements. Gene expression pattern analysis showed that the selected genes had different responses to drought, high salt and low temperature stress. Most of the genes played key roles under drought and salt stress, and the response patterns were more similar. The VIGS method was used to silence the *SLmTERF13* gene, which was significantly upregulated under drought and salt stress, and it was found that the resistance ability of silenced plants was decreased under both kinds of stress, indicating that the *SLmTERF13* gene was involved in the regulation of the tomato abiotic stress response. These results provide important insights for further evolutionary studies and contribute to a better understanding of the role of the *mTERF* genes in tomato growth and development and abiotic stress response, which will ultimately play a role in future studies of tomato gene function.

## 1. Introduction

The most obvious difference between eukaryotes and prokaryotes is the presence or absence of organelles. Most of the organelle genes in chloroplasts and mitochondria have been transferred to the nucleus or lost during evolution from their bacterial progenitors in different plant species; nowadays, only a few genes related to photosynthesis, electron transport chains, and gene expression remain in chloroplasts and mitochondria [1,2]. Most of the proteins contained in mitochondria and chloroplasts are encoded by nuclear genes, and organellar gene expression (OGE) requires the regulation of nuclear gene proteins. Mitochondrial transcription termination factor (mTERF) is a DNA-binding protein encoded by a nuclear gene, contains an mTERF motif (composed of 30 tandem repeats of amino acid residues, including three leucine zipper-like structures), and functions in mitochondria [3]. This protein can combine with mitochondrial nucleic acids and plays a regulatory role in mitochondrial gene replication, transcription and translation [4]. By separating and purifying human mitochondrial lysates, Kruse et al. [5] first discovered mTERF, which can promote the termination of mitochondrial DNA (mtDNA) transcription. Since then, more *mTERF* genes have been found in animals and plants. In metazoans and plants, mTERF can be divided into four subfamilies, named mTERF1–mTERF4 [3,6]. But there are more *mTERF* gene family members in terrestrial plants than in animals, with approximately 30 members currently present in the former [4,7]. For example, 35 *mTERFs* have been identified in Arabidopsis and pepper, and 33, 31, and 25 *mTERFs* exist in rice, corn, and grape, respectively [8,9,10,11].

Members of the mTERF family of plants can regulate the function of mitochondrial and chloroplast genes and play an important role in biological evolution, plant growth and development, and stress resistance, functioning in mitochondria and chloroplasts [3,12,13]. For example, Chlamydomonas contains a family of MOC proteins (MOC1~MOC6) with characteristics similar to those of mTERF. One study found that if *MOC1* was missing, the stability of the mitochondrial respiratory chain complex was disrupted, leading to termination of mtDNA transcription [14]. In Arabidopsis, *mTERF5* and *PED191* (*mTERF6*) are in chloroplasts. The deletion of *mTERF5* enhances the tolerance of plants to salt stress, weakens the sensitivity of plants to abscisic acid (ABA), and lightens the color of plant leaves, stems, and sepals [15]. Early chloroplast development of *PED191* mutants is hindered, resulting in albinism of seedlings and seedling death [16,17]. Deletion of *mTERF18*, which localizes in mitochondria, leads to the enhanced heat tolerance of *Arabidopsis thaliana* plants and increases the expression of multiple stress response genes, and the leaves become dark green [18]. *Zm-TERF4* in the chloroplast matrix of maize has been shown to react with multiple introns in chloroplast genes by immunoprecipitation. The deletion of this gene leads to the deletion of ribosomes in the plastids, and homozygous *Zm-TERF4* mutants die when they reach the 3~4-leaf stage [19].

Tomato has rich nutritional value and unique flavor and is of the horticultural crop varieties, and the fruit contains a variety of vitamins, carotenoids, phenolic amino acids, and minerals [20]. In addition, tomato has high economic value and medicinal value, with anti-inflammatory, antioxidant, anti-cancer, and other effects and has been widely cultivated around the world [21,22]. *mTERF* plays an important role in plant growth, development, and stress response. At present, the *mTERF* gene family has been studied in Arabidopsis, maize, and other crop species. However, whether *mTERF* gene can have a regulatory effect on growth and development and stress in tomato remains to be studied. Therefore, in this study, the whole genome of tomato was mined for *mTERF* gene family members using bioinformatics methods, and relevant structure and function prediction analyses of the family members were carried out. Moreover, through the analysis of the expression pattern of the stress response and the preliminary functional study of key response genes, the role of this family gene in the regulation of the tomato stress resistance response was clarified, which laid the foundation for the application of these family members in the genetic improvement of tomato stress resistance.

## 2. Results

### 2.1. Identification of mTERF Gene Family Members and Construction of an Evolutionary Tree of Tomato

Based on HMM and structure domain analysis, a total of 28 *mTERF* genes were identified, named *SLmTERF1*–*SLmTERF28*, and the basic information of these gene family members is shown in Table 1. As shown in the table, the sequence length of mTERF proteins is between 202 (*SLmTERF19*) and 1222 (*SLmTERF20*) amino acids. The relative molecular weight of the proteins ranges from 24,224.60 (*SLmTERF19*) to 134,350.96 (*SLmTERF20*) Da, and the theoretical isoelectric point ranges from 5.67 to 9.78. The minimum aliphatic amino acid index is 38.02 (*SLmTERF20*), and the maximum is 110.59 (*SLmTERF25*). The analysis of the instability coefficient of encoded proteins showed that 11 mTERFs were stable proteins (instability coefficient < 40), and the rest were unstable proteins. The results of the neural algorithm network (Figure S1) showed that only the SLmTERF3 protein had a signal peptide sequence, and the splicing site was most likely located at amino acid positions 18–19, indicating that the SLmTERF3 protein may play a role in signal recognition in transmembrane transport.

**Figure 1:**
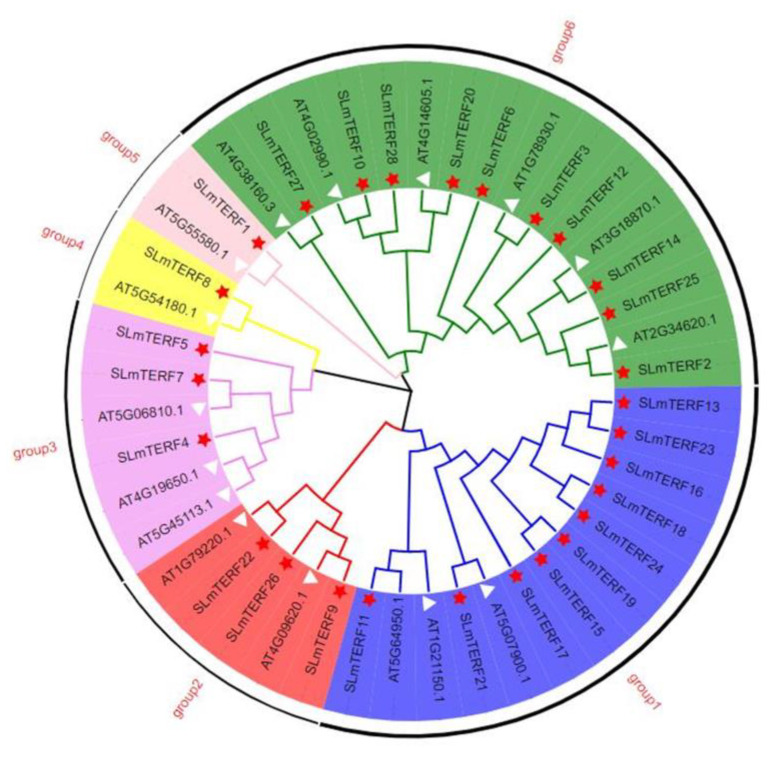
Phylogenetic tree of *mTERF* in *Arabidopsis thaliana* and tomato. The phylogenetic tree was constructed using the NJ method, with 1000 bootstrap replications. The 45 *mTERFs* were divided into 6 groups: blue, red, purple, yellow, pink, and green. The red five-pointed stars and white triangles represent tomato and Arabidopsis, respectively.

To explore the evolutionary relationship between the *mTERF* gene families of tomato and Arabidopsis, MEGA 7 software was used to construct a phylogenetic tree (Figure 1). As shown in the figure, 45 *mTERFs* were divided into six groups, named groups 1–6. Among them, group 1 and group 6 had the largest number of *SLmTERF* members—10. Group 2 and group 3 contained three *SLmTERFs*. Group 4 and group 5 each contained only one *SLmTERF*.

### 2.2. mTERF Gene Structure, Protein Secondary Structure, and Motif Analysis in Tomato

**Figure 2:**
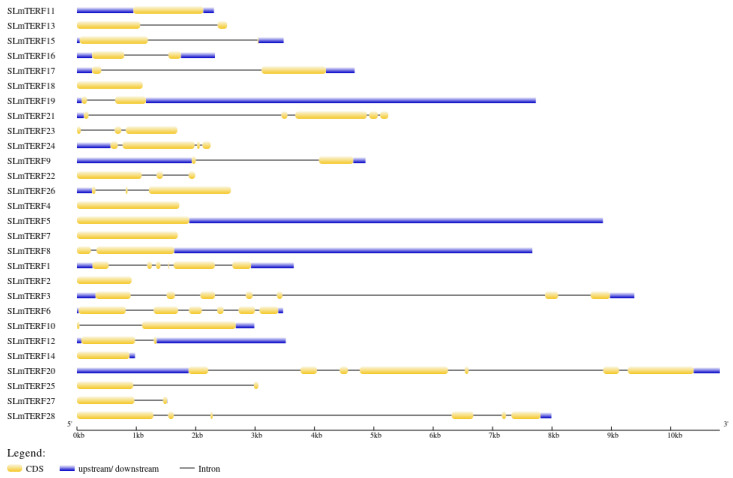
Structure of the *mTERF* gene in tomato. CDSs, upstream and downstream coding regions, and introns are represented by yellow, purple, and black lines, respectively.

**Figure 3:**
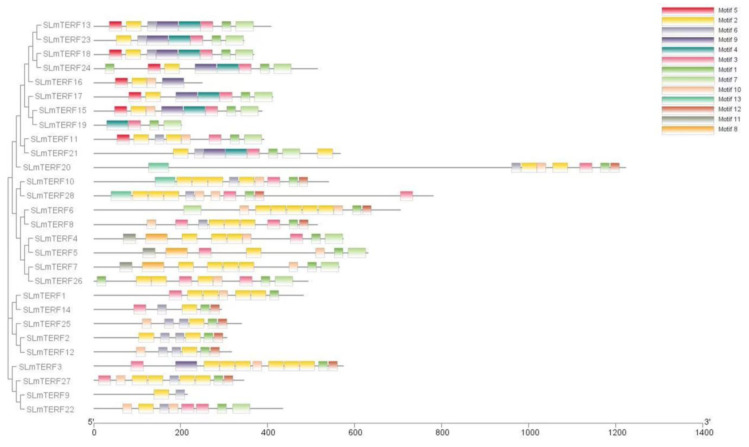
Motif analysis of *SLmTERFs*. The motif types are represented by different colored squares.

The results of gene structure mapping are shown in Figure 2. The number of exons in the 28 tomato *mTERF* genes ranged from 1 to 7. Only *SLmTERF2*, *SLmTERF4*, *SLmTERF5*, *SLmTERF7*, *SLmTERF11*, *SLmTERF14*, and *SLmTERF18* have no introns, and the number of introns in genes with introns ranges from 1 to 6. The results of protein secondary structure analysis are shown in Table 2. Tomato mTERF proteins have α helices, extended chains, and random coils, among which α helices and random coils account for the largest proportion. The tomato mTERF protein conserved motif information is shown in Figure 3. As shown in the figure, a total of 13 motifs were identified in tomato mTERFs. Among them, the members of the G3 group all have motif 1, motif 2, motif 7, motif 8, and motif 11.

### 2.3. Chromosome Localization Prediction, Subcellular Localization, and Cis-Acting Element Analysis

**Figure 4:**
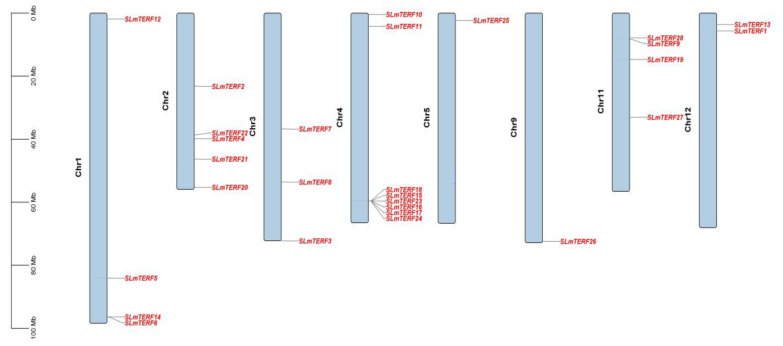
Chromosomal mapping of the *mTERF* gene in tomato. The chromosome number is located at the top of each chromosome, and the scale value on the left corresponds to the chromosome length.

**Figure 5:**
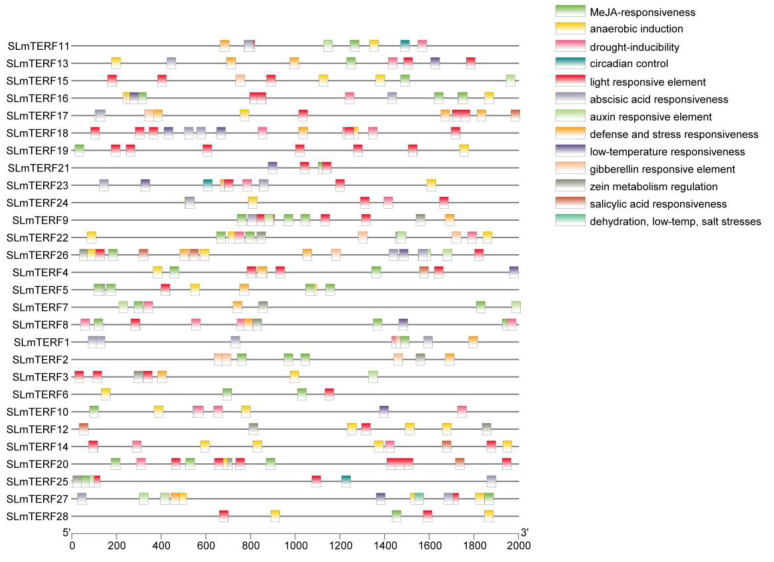
Cis-acting elements of the *mTERF* genes in tomato. The different colored squares represent different cis-acting elements.

The chromosome localization results are shown in Figure 4; the 28 tomato *mTERF* genes showed uneven distribution across the chromosomes. Chromosome 4 contained the largest number of genes—a total of eight—and showed coaggregation, which may be related to the tandem duplication of chromosomes; chromosome 5 and chromosome 9 each contained one gene; chromosome 1 and chromosome 11 had four genes; and chromosome 2, chromosome 3, and chromosome 12 contained five, three, and two genes, respectively. As shown in Table S1, a total of 15 SLmTERF proteins were in mitochondria or chloroplasts, and these SLmTERFs may play a role in regulating mitochondrial and chloroplast genes. In addition, the remaining 13 SLmTERF proteins were in other locations. The specific location of these proteins needs to be verified by further experiments. Figure 5 shows the analysis results of cis-acting elements. As shown in the figure, the members of the tomato *mTERF* gene family contain abiotic stress-responsive elements such as those corresponding to light, salt stress, drought, and low temperature. In addition, it also has responsive elements such as hormones (ABA, GA, MeJA, IAA), circadian rhythm, defense, and stress. The results indicated that tomato *mTERFs* may play a regulatory role in tomato growth and development and the response to abiotic stress.

### 2.4. Tissue Specificity Analysis of the mTERF Gene in Tomato

**Figure 6:**
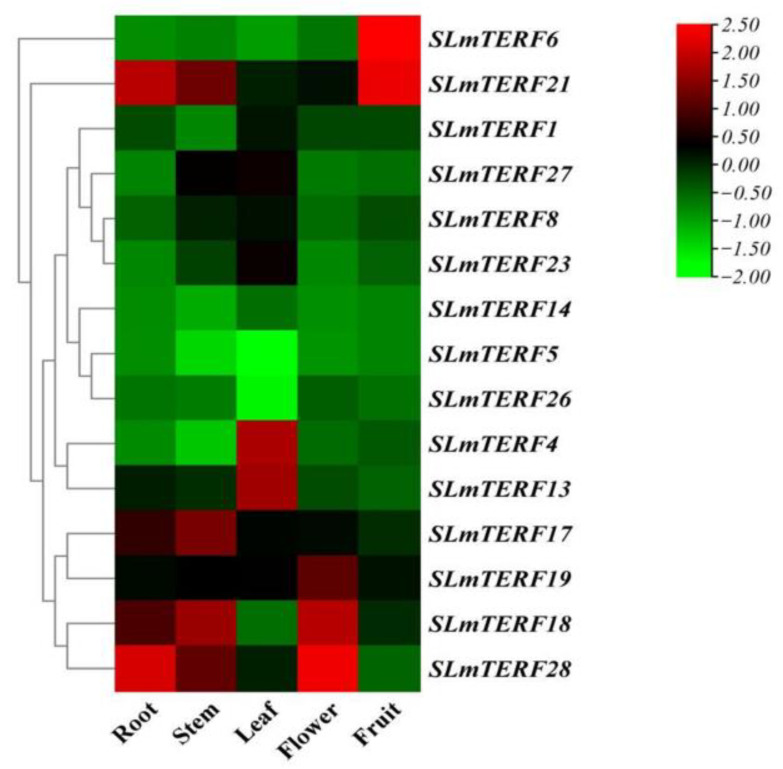
Expression of 15 *SLmTERF* genes in different tissues. The chromaticity on the right side of the heatmap shows the relative expression, and the color gradient from green to red corresponds to an increase in expression.

The specific expression of genes at different stages of plant growth regulates the normal growth and development of plants. Figure 6 shows the expression heatmap of 15 typical *SLmTERF* genes in different tissues constructed. These 15 genes were expressed to different degrees in the five tissues, and the differences were significant. Compared with other genes, *SLmTERF6* and *SLmTERF21* were expressed significantly in tomato fruit. The expression of *SLmTERF28* was the highest in the flowers. *SLmTERF21* and *SLmTERF28* were highly expressed in the roots. The expression of *SLmTERF4* and *SLmTERF13* in the leaves was significant. The expression of *SLmTERF17* and *SLmTERF18* in the stems was weakly significant. We hypothesize that these significantly expressed *mTERF* genes maintain normal plant growth and development by affecting mitochondrial or chloroplast gene replication, transcription, or translation in different tissues.

### 2.5. Analysis of mTERF Gene Expression in Tomato under Abiotic Stress

**Figure 7:**
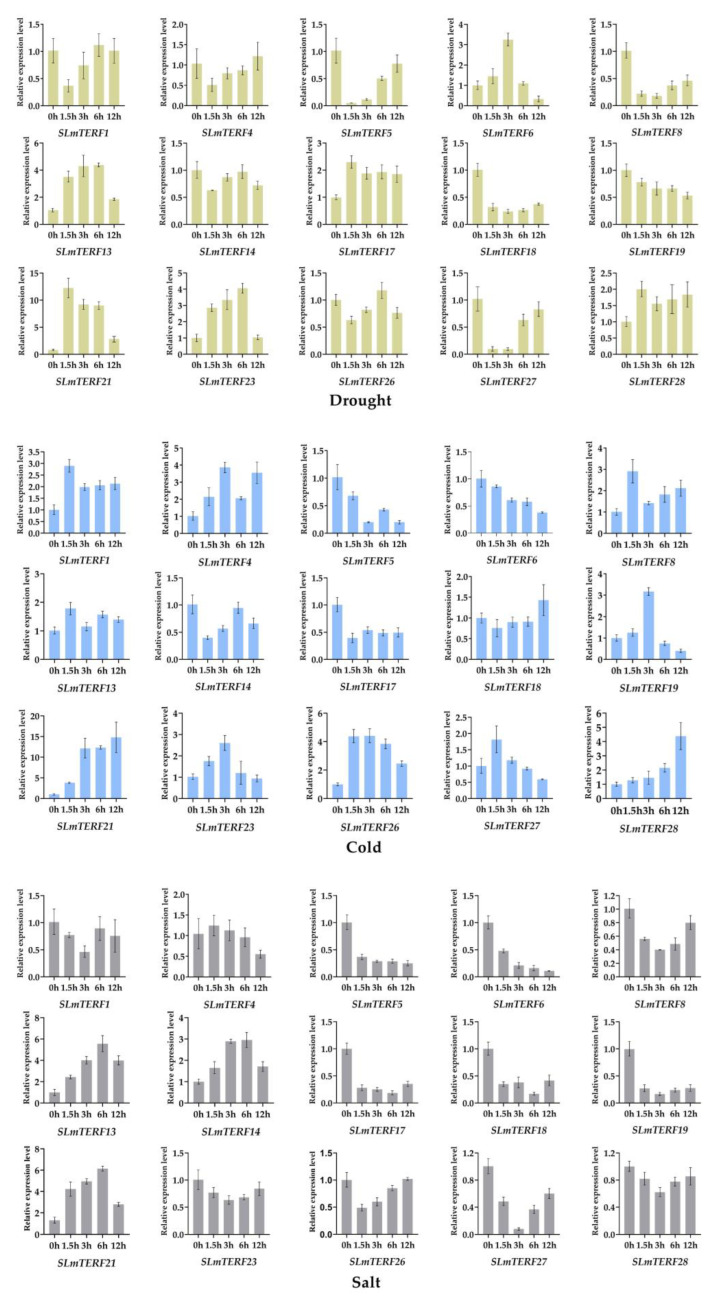
Under abiotic stress conditions, the expression levels of 15 *SLmTERF* genes changed during different periods.

The 15 typical *SLmTERF* genes screened on the basis of their phylogenetic relationships were subjected to qRT-PCR to analyze the changes in the expression of the tomato *mTERF* gene family members when the plants were under abiotic stress conditions (Figure 7). The expression levels of six genes were upregulated under drought stress, and the expression levels of three genes, *SLmTEF13*, *SLmTERF21*, and *SLmTERF23*, were significantly upregulated. After cold stress treatment, a total of nine genes were upregulated, of which the expression of *SLmTERF21* and *SLmTERF26* increased significantly. However, under salt stress, the expression of most genes showed a downward trend, but the expression of *SLmTERF13* and *SLmTERF21* increased significantly. *SLmTERF13*, whose expression was significantly upregulated under drought and salt stress, was selected for subsequent functional verification.

### 2.6. Cloning and Subcellular Localization of the SLmTERF13 Gene

**Figure 8:**
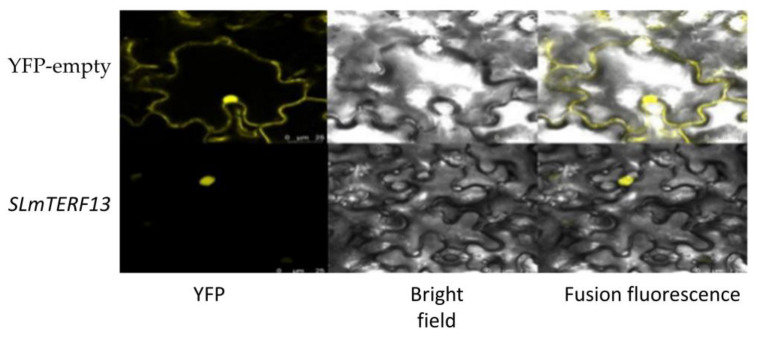
Results of *SLmTERF13* subcellular localization.

Homologous cloning and sequencing revealed that the *SLmTERF13* gene sequence was completely consistent with the sequence in the database, and the comparison rate reached 100%. Further subcellular localization experiments showed that the gene was localized in the nucleus (Figure 8).

### 2.7. Functional Analysis of the SLmTERF13 Gene

#### 2.7.1. Phenotypic Analysis of Stress Resistance of Gene-Silenced Plants

**Figure 9:**
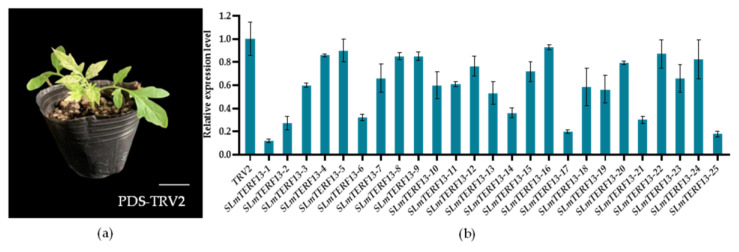
Detection of gene-silenced plants. (**a**) PDS-silenced indicator plants exhibit albino phenomena. (**b**) Expression levels in *SLmTERF13*-silenced plants.

After albino PDS-silenced indicated plants (Figure 9a), plants with more than a 50% reduction in target gene expression (Figure 9b) were selected for drought and salt stress treatments.

**Figure 10:**
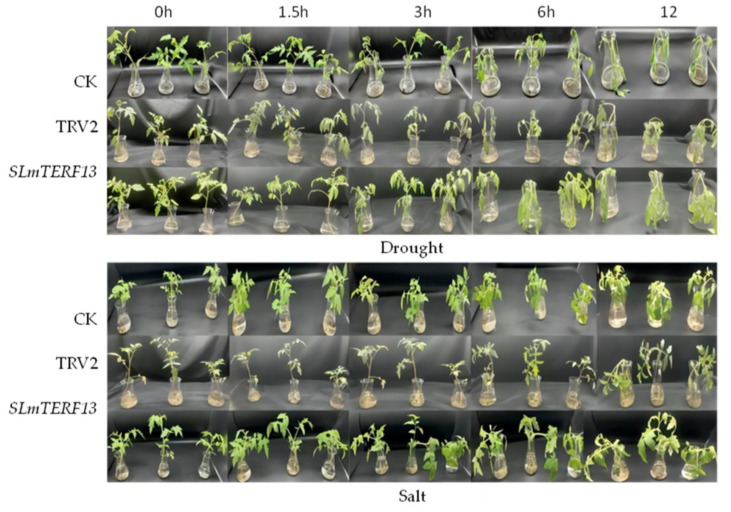
Phenotypic changes in plants under abiotic stress at different periods.

The phenotypic changes after treatment are shown in Figure 10. With prolonged treatment time, the degree of plant damage gradually increased. However, the occurrence time of leaf wilting and stem bending of the *SLmTERF13*-silenced plants was earlier than that in the control plants, and the degree of plant damage at each time point was more obvious than that of the control plants.

#### 2.7.2. Physiological Index Measurements

**Figure 11:**
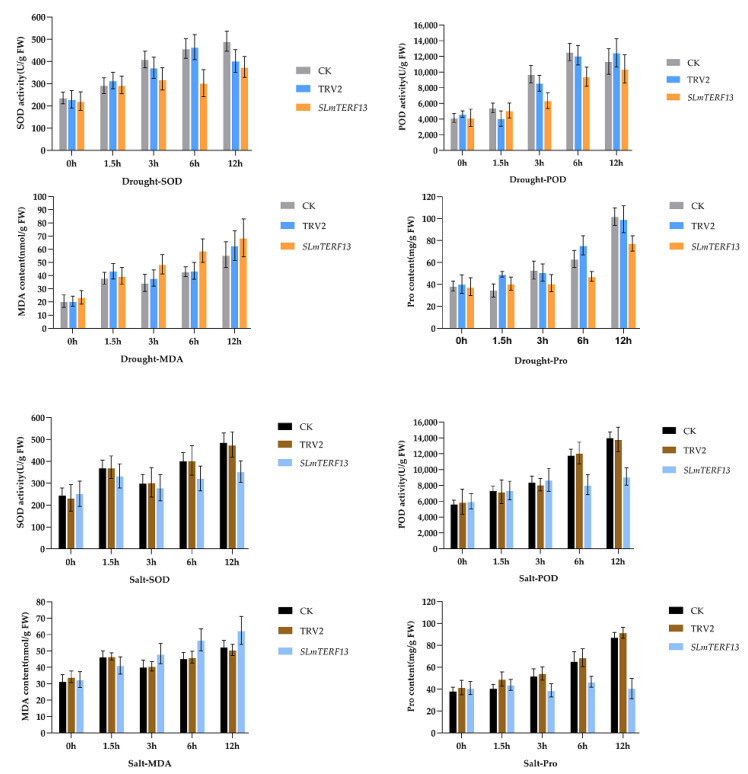
Under abiotic stress, SOD and POD activities and MDA and Pro contents changed at different time points.

The physiological indexes after 12 h of abiotic stress are shown in Figure 11. As shown in the figure, SOD activity, POD activity, Pro content, and MDA content all showed an increasing trend. Except for the MDA content, which was significantly higher than that of the control group, the other values of *SLmTERF13*-silenced plants were significantly lower than those of the control group.

#### 2.7.3. Active Oxygen Staining

**Figure 12:**
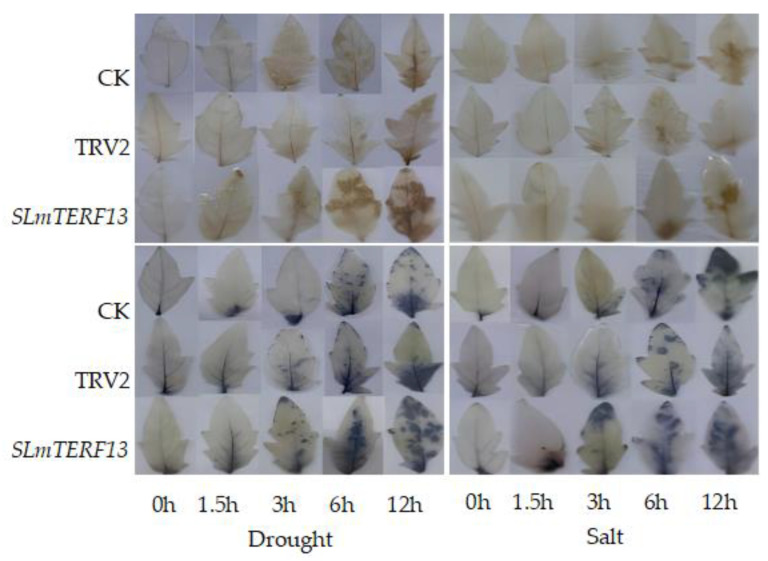
Changes in DAB and NBT staining of plant leaves at different time points under abiotic stress. The top image shows DAB staining, and the bottom image shows NBT staining.

Figure 12 shows the results of DAB and NBT staining under drought and salt stress.

As time progressed, the staining area of the leaves of the three groups of tomato plants gradually increased, and the staining degree gradually intensified. However, compared with the control plants, the *SLmTERF13*-silenced plants had a larger staining area and more intense degree of staining at the same time points.

#### 2.7.4. Chlorophyll Content and Electrical Conductivity Determination

**Figure 13:**
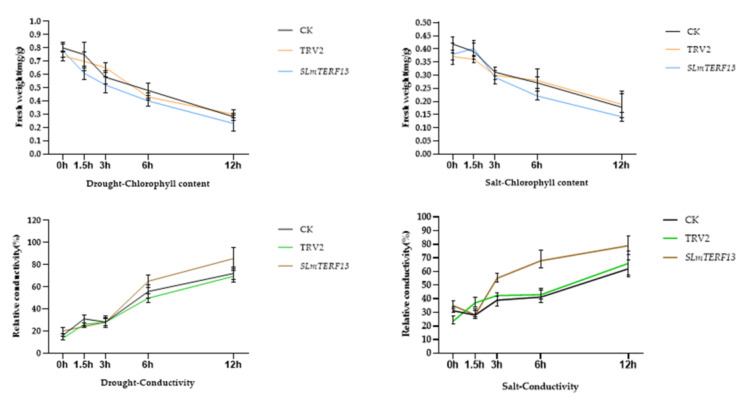
Chlorophyll content and electrical conductivity changes at different time points during abiotic stress.

As shown in Figure 13, compared with the control, in *SLmTERF13*-silenced plants, the degree of decrease in chlorophyll content was slightly higher than that in the control group, but the increase in electrical conductivity was significantly higher.

## 3. Discussion

A total of 28 members of the tomato mTERF family were identified in this study. There were 35 genes of this family in *Arabidopsis thaliana*, and 35, 33, 31, and 25 genes in capsicum, rice, maize, and grape, respectively, indicating that the family number of genes in different species was similar, and the number of family members had little relationship with the genome size of species. The genes of this family in tomato can be divided into six groups, but the distribution of the number of genes in the six groups is quite different. Group 1 and Group 6, which have more members, are closer to the end of evolution, indicating that the genes of this family have a significant bias in the process of evolution. This phenomenon also appears in the genes of the same family in Arabidopsis. This suggests that this evolutionary trend may be common among different plants.

Cis-acting elements are important molecular switches that play an important role in plant growth and development and abiotic stress responses through their participation in transcriptional regulation [23,24,25]. In this study, 28 *mTERF* gene family members all contained different cis-acting elements, including 13 types related to abiotic stress, hormones, and other related response elements. Abscisic acid response elements (ABREs), low temperature response elements (LTREs), drought response elements (DREs), etc., have been shown to be closely related to stress resistance. Twelve genes, such as *SLmTERF1*, *SLmTERF9*, *SLmTERF11*, and *SLmTERF13*, contain ABREs, and reports have shown that cis-acting elements play a role in regulating osmotic stress and cold stress in ABA-dependent genes [26]. For example, the *RAB16* gene containing ABREs in rice is expressed in late embryogenetic seeds and in vegetative tissues induced by ABA and osmotic stress [27,28]. *RAB16* enhances the stress resistance of rice by encoding proteins related to osmotic stress or other protective effects. *SLmTERF4*, *SLmTERF8*, *SLmTERF10*, *SLmTERF13*, a total of 10 genes contain LTRE. Genes containing LTRE play a key role in the regulation of plant low temperature. For example, in Brassica napus, mutation of the core pentamer CCGAC in the LTRE in the 5′-proximal region of the *BN115* gene affects the low-temperature regulation expression of *BN115* [29]. The presence of these cis-acting elements provides a structural basis for the participation of these *SLmTERF* genes in the regulation of stress resistance.

To further explore the response rules of the tomato *mTERF* gene family to different stresses, we analyzed the expression patterns of the tomato mTERF gene family under drought, salt and low temperature stresses. The results showed that all the selected genes had different responses under different stresses, and most of the genes showed different response modes under the three stresses. For example, *SLmTERF17* was upregulated under drought stress and downregulated under salt stress and cold stress; *SLmTERF6* was slowly downregulated under cold stress and rapidly downregulated under salt stress but was first upregulated and then downregulated under drought stress. By association evolutionary relationship analysis, we found that the family members divided into the same group had completely different response rules, indicating that the structural evolution of the family genes had little relationship with functional selection. Further analysis combined with cis-acting elements showed that the stress response patterns of multiple genes were consistent with the prediction of their component functions, but there were also some genes, such as *SLmTERF21*, that had no drought response-related elements but were significantly upregulated under drought stress. This suggests that genes in this family may also be involved in the regulation of the stress response through other signals or indirect pathways. In all the analysis of expression patterns, we found that *SLmTERF1*, *SLmTERF21*, and *SLmTERF17* showed significant expression changes in the early stage of cold stress, drought stress, and salt stress, respectively; *SLmTERF28* showed a significant increase in the late stage of cold stress; *SLmTERF23* showed a sharp increase in the early stage and a sharp decline in the later stage under drought stress. This phenomenon indicates that tomato mTERF family members may play a role in different stages of stress resistance regulation.

For *SLmTERF13*, which was significantly upregulated under drought and salt stress, VIGS technology was used for preliminary functional verification. The results showed that the downregulation of the gene led to a decrease in the drought and salt tolerance of tomato plants. When the physiological indexes of *SLmTERF13*-silenced plants were detected, it was found that the changes in SOD and POD activities and MDA and Pro contents of *SLmTERF13*-silenced plants were similar under drought stress and salt stress, but the changes in chlorophyll content and electrical conductivity were obviously different in the early stage of the two stresses; but the trends were consistent in the later stage. These results suggest that the *SLmTERF13* gene may have some differences in the regulation of drought and salt stress responses, but both play positive regulatory functions in the end. Due to the limitations of VIGS, the functional verification method for *SLmTERF13* in this study and the process of this gene’s participation in drought and salt stress should be further explored in detail to further clarify the similarities and differences in the regulation of this gene under different adversities. In this study, *SLmTERF13* was in the nucleus, which is consistent with the localization prediction of this gene and the localization characteristics of this gene family. Previous studies have shown that most of the genes in this family are encoded in the nucleus and then transferred to the chloroplast or mitochondria for function. In this study, the chlorophyll content of plants silenced by *SLmTERF13* did not change significantly compared with that of control plants, but the chlorophyll content of plants silenced by *SLmTERF13* decreased more during the whole process after stress, indicating that the decrease in the expression of this gene would not affect chlorophyll accumulation under normal circumstances but would aggravate chlorophyll loss when stress occurred. Combined with the upregulated expression trend of this gene under drought and salt stress, we speculated that *SLmTERF13* may reduce chlorophyll loss and maintain chlorophyll content balance through some positive regulatory pathway and then positively regulate the resistance of tomato plants.

## 4. Materials and Methods

### 4.1. Identification of mTERF Gene Family Members and Construction of an mTERF Evolutionary Tree in Tomato

The hidden Markov model (HMM) of the *mTERF* conserved domain (numberPF02536) was obtained from the Pfam database (http://pfam.xfam.org/family/ (accessed on 1 June 2020)) to screen candidate genes [30]. The nucleotide sequence and amino acid sequence (https://sgn.cornell.edu/help/index.pl/ (accessed on 5 June 2020)) of 28 candidate tomato *mTERF* genes were downloaded from the Ensembl Plants database (http://plants.ensembl.org/index.html/ (accessed on 7 June 2020)) for subsequent analysis [31]. The conserved domains of 28 tomato *mTERF* genes were verified by the SMART database (http://smart.embl-heidelberg.de/ (accessed on 12 June 2020)).

Thirty-five Arabidopsis mTERF protein sequences were obtained from The Arabidopsis Information Resource (TAIR) (http://www.arabidopsis.org/ (accessed on 15 June 2020)) [32]. The mTERF protein sequences of tomato and Arabidopsis were subjected to multiple sequence alignment via ClusterX. Based on the comparison results, MEGA 7 was used to construct a rootless developmental tree using the neighbor-joining (NJ) method (bootstraps = 1000) [33].

### 4.2. Analysis of the Physical and Chemical Properties of Tomato mTERF Proteins

The amino acid number, relative molecular weight, and theoretical isoelectric point of the mTERFs were predicted by the ProtParam tool (https://web.expasy.org/protparam/ (accessed on 21 June 2020)) of ExPASy [34]. SignalP 4.1 (http://www.cbs.dtu.dk/services/SignalP/ (accessed on 23 June 2020)) was used to predict signal peptides of 28 tomato mTERF proteins [35].

### 4.3. Gene Structure, Protein Secondary Structure, and Motif Analysis of Tomato mTERFs

The full-length sequences and CDSs of the 28 tomato *mTERF* genes were imported into the Gene Structure Display Server (GSDS) (http://gsds.gao-lab.org/index.php (accessed on 28 June 2020)) for gene structure analysis [36]. The 28 tomato mTERF protein sequences were submitted to Prabi (https://npsa-prabi.ibcp.fr (accessed on 3 July 2020)) and MEME (https://meme-suite.org/meme/ (accessed on4 July 2020)) for protein secondary structure and motif analysis, respectively [37].

### 4.4. Chromosome Localization Prediction, Subcellular Localization, and Cis-Acting Element Analysis of Tomato mTERFs

The annotation information of 28 tomato *mTERF* genomes was retrieved by SGN (https://sgn.cornell.edu/help/index.pl (accessed on 11 July 2020)), and the position of tomato *mTERF* on chromosomes was obtained by MG2C (http://mg2c.iask.in/mg2c_v2.0/ (accessed on 11 July 2020)). The subcellular location of tomato mTERF was predicted by TargetP-2.0 (http://www.cbs.dtu.dk/services/TargetP (accessed on 18 July 2020)) combined with 28 tomato mTERF protein sequences. From the tomato genome database (https://solgenomics.net/ (accessed on 20 July 2020)), the 2000 bp upstream promoter sequences of the 28 candidate genes were downloaded and submitted to Plant-CARE (http://bioinformatics.psb.ugent./webtools/plantcare/html/ (accessed on 21 July 2020)) for the prediction of cis-acting elements in the promoter regions.

### 4.5. Plant Materials and Treatment Methods

The tomato variety “Ailsa Craig” and the tobacco variety “Nicotiana benthami-ana” were used in this experiment. The above experimental materials were provided by the Tomato Research Institute of Northeast Agricultural University.

“Ailsa Craig” plants were grown in a greenhouse (13 h light, 26 °C temperature, 45% relative humidity). Tomato seedlings exhibiting the same growth were selected and subjected to low temperature (4 °C), drought (15% PEG6000), or salt (0.2 mol/L NaCl). At 0, 1.5, 3, 6, and 12 h after the abiotic stress treatments, 3–4 leaves of the tomato seedlings were randomly sampled (three biological replicates each). The samples were frozen in liquid nitrogen and stored at −80 °C. The samples were used for gene expression level analysis.

“Ailsa Craig” plants were grown in a greenhouse (11 h light, 21 °C temperature, 45% relative humidity). When the tomato fruits were ripe, the roots, stems, leaves, flowers and fruits were sampled, and three biological replicates were collected. The samples were used for tissue-specific expression analysis.

The gene silencing material “Ailsa Craig” and the subcellular localization material “Nicotiana benthamiana” were grown in a greenhouse (13 h light, 26 °C, 45% relative humidity).

### 4.6. cDNA Synthesis and qRT-PCR

RNA extraction from tomato leaves. The kit selected for RNA extraction was an RNA mini kit (Watson, China). The synthesized cDNA was reverse transcribed into RNA, and the reverse transcription kit was sourced from Beijing TransGen Biotech. qRT-PCR was used for gene expression analysis and tissue-specific expression analysis of 15 *SLmTERFs* selected after phylogenetic tree relationships were combined. NCBI online software (https://www.ncbi.nlm.nih.gov/tools/primer-blast/ (accessed on 6 September 2020)) was used to design primers for the 15 *SLmTERF* genes (Table S2), with tomato *Actin-7* as the internal reference [38,39]. The reaction mixture for fluorescence quantification was 20 µL, comprising 10 µL of SYBR master mix, 0.5 µL of forward and reverse primers, 1 µL of cDNA template, and 8 µL of ddH_2_O. The qRT-PCR protocol was as follows: 95 °C for 30 s, 95 °C for 10 s, 60 °C for 30 s, 95 °C for 15 s, and 60 °C for 60 s, for which there were 40 cycles. Gene expression was calculated using the 2^−ΔΔCt^ method [40].

### 4.7. Gene Silencing and Stress Resistance Treatments in Silenced Plants

*SLmTERF13* gene silencing fragments were designed using the SGN-Vigs tool (http://vigs.solgenomics.net/ (accessed on 15 January 2021)) [41], and the gene silencing primer information can be found in Table S2 for details. After PCR amplification of the gene silencing fragments, the target gene and plasmid were digested with EcoR1 and BamH1. The target fragment and the TRV2 plasmid were reconstituted using T4 ligase, the recombinant plasmid was transformed into DH5α *Escherichia coli*, and the plasmid was extracted using the bacterial solution that was successfully sequenced. Finally, the extracted plasmid was transferred to *Agrobacterium* GV3101 for culture for the infection experiment.

The bacterial solutions of TRV2 empty vector-containing, *PDS*-TRV2 and *SLmTERF13*-TRV2, were used to infect the leaves of tomato seedlings with the same growth and free of diseases and pests using a 1ml syringe. High-moisture conditions in the dark for 72 h were used to promote infection, and then the leaves were incubated in a room with a temperature of 21 °C, a photoperiod of 15 h, and a relative humidity of 70%. Each group consisted of 35 infected tomato seedlings, and three biological repeats were carried out. The gene silencing system was suggested to be effective when the tomato seedlings infected with *PDS*-TRV2 were albino. Afterward, the leaves of the *SLmTERF13* gene-silenced plants were removed to detect the silencing efficiency by qRT-PCR.

Since the phenotypes of the gene-silenced plants and the control plants were not significantly different under the cold stress environment for 12 h, subsequent tests were conducted only under drought and salt stress. The selected gene-silenced tomato plants were divided into a drought group (15% PEG6000) and a salt stress group (200 mol/L NaCl), and wild-type and TRV2 empty vector-containing plants were used as controls. The plant phenotypes were observed, and leaf samples were taken at 5 time points (0, 1.5, 3, 6, and 12 h). The leaves were subsequently stored at −80 °C. Each treatment was repeated three times.

### 4.8. Physiological Index Measurements

#### 4.8.1. Determination of Superoxide Dismutase (SOD) and Peroxidase (POD) Activities and Proline (Pro) and Malondialdehyde (MDA) Contents

The activities of SOD and POD and the contents of Pro and MDA in the leaves of wild-type plants, TRV2 empty vector-containing plants, and *SLmTERF13* gene-silenced plants were measured according to the operation steps of the kits used (Keming, China).

#### 4.8.2. Reactive Oxygen Staining Observation

Leaves of wild-type plants, TRV2 empty vector-containing plants, and *SLmTERF13* gene-silenced plants were immersed in 3,3′-diaminobenzidine (DAB) and nitroblue tetrazolium (NBT) dye solution. After incubating in the dark for 12 h, the dye solution was discarded, 30 mL of absolute ethanol was added, and the materials were heated in a 100 °C water bath for 12 min while being shaken every 3 min. If the color of the leaves had not completely faded, they were washed again with absolute ethanol several times. The completely faded leaves were placed on a slide to observe the staining and collect images.

#### 4.8.3. Determination of Chlorophyll Content and Electrical Conductivity

The chlorophyll content was determined using a chlorophyll assay kit (Solarbio, China). One gram of leaf tissue of wild tomato, TRV2 empty vector-containing tomato, and silenced tomato plants were weighed and placed in a centrifuge tube filled with 25 mL of sterile water. After being sealed and soaked for 16 h in the dark, the initial electrical conductivity of the solution was measured using a conductance meter. Then, the centrifuge tube was heated in a water bath for 30 min and allowed to cool to room temperature to determine the final conductivity of the solution. The relative conductivity = initial conductivity/final conductivity × 100% [42]. Three replications were performed, and the average value was taken.

### 4.9. Subcellular Localization of SLmTERF13 Protein

The YFP-*SLmTERF13* primer was designed using NCBI software (Table S3), and then Primer Premier 5.0 was used to detect whether the designed primer sequence contained the restriction sites of BamH I and Kpn I. After detection, Beijing Tsingke Biotech Co., Ltd. (Beijing, China) was commissioned to conduct primer synthesis. The methods of cutting target gene and plasmid, recombining target fragment with plasmid, transferring recombinant plasmid into *Escherichia coli*, transferring plasmids into *Agrobacterium*, and infecting tobacco plants were similar to 4.7. After 3 days of cultivation in the dark, the leaf area was torn off, and fluorescence imaging was performed by laser coaggregation microscopy.

## 5. Conclusions

**Figure 14:**
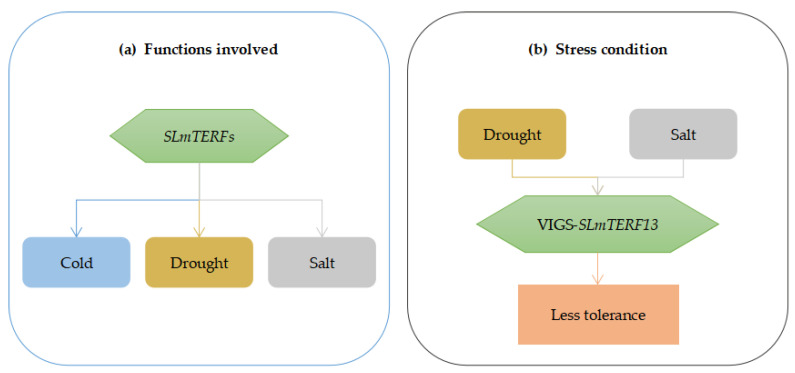
(**a**) Functions involved by the *SLmTERFs* gene family. (**b**) *SLmTERF13* gene silencing decreased plant resistance to drought and salt stress.

This study analyzed the tomato *mTERF* gene family at the whole-genome level, revealing a total of 28 *SLmTERFs* that were then divided into six groups. Then, the phylogenetic relationships, physicochemical properties, gene structures, chromosome positions, cis-acting elements, and conserved motifs were characterized using bioinformatics methods. The expression of 15 *SLmTERFs* was analyzed by qRT-PCR, and it was found that most genes could respond to abiotic stresses(Figure 14a). Afterward, the function of the *SLmTERF13* gene was verified by gene silencing under stress conditions, and it was found that *SLmTERF13* had a positive regulatory effect under salt stress and drought stress (Figure 14b). Finally, the *SLmTERF13* gene was located in the nucleus using subcellular localization. These results can help us further explore the regulatory mechanism of tomato *mTERF* gene family members in response to abiotic stress and provide a foundation for the genetic improvement of tomato resistance.

## Figures and Tables

**Table 1:** Database number, gene name, amino acid number, relative molecular weight, theoretical isoelectric point, aliphatic amino acid index, and instability coefficient of the tomato *mTERF* gene family.

GENE ID	GENE NAME	Number of Amino Acids (aa)	Molecular Weight (Da)	Theoretical pI	Aliphatic Index	GRAVY	Instability Index
Solyc12g015640.2.1	*SLmTERF1*	482	56,021.91	9.23	87.34	−0.494	44.09
Solyc02g021430.1.1	*SLmTERF2*	306	34,876.53	8.78	93.33	−0.148	46.43
Solyc03g124040.3.1	*SLmTERF3*	574	65,951.33	9.18	97.65	−0.090	52.19
Solyc02g069320.1.1	*SLmTERF4*	574	65,300.50	8.76	96.11	−0.147	35.39
Solyc01g090500.3.1	*SLmTERF5*	630	72,679.44	8.18	88.83	−0.133	46.50
Solyc01g109630.3.1	*SLmTERF6*	705	81,980.16	8.96	89.67	−0.283	45.02
Solyc03g063390.1.1	*SLmTERF7*	565	65,319.42	9.12	101.42	−0.107	40.83
Solyc03g081300.3.1	*SLmTERF8*	514	57,846.31	7.65	103.17	0.015	42.70
Solyc11g017430.2.1	*SLmTERF9*	215	24,380.79	6.82	79.30	−0.385	48.39
Solyc04g005630.3.1	*SLmTERF10*	540	61,573.69	7.61	97.22	−0.150	45.02
Solyc04g011700.2.1	*SLmTERF11*	392	44,616.77	9.78	107.09	0.017	38.51
Solyc01g007750.3.1	*SLmTERF12*	317	36,039.24	9.18	95.52	−0.008	59.20
Solyc12g010650.1.1	*SLmTERF13*	407	47,343.82	9.59	102.65	−0.046	26.94
Solyc01g109550.2.1	*SLmTERF14*	294	34,528.11	9.32	87.55	−0.233	45.58
Solyc04g072510.3.1	*SLmTERF15*	387	43,999.61	9.17	105.99	0.015	35.54
Solyc04g072530.2.1	*SLmTERF16*	250	28,271.08	9.28	102.88	0.130	30.46
Solyc04g072540.3.1	*SLmTERF17*	412	46,777.19	9.77	103.11	0.025	37.75
Solyc04g072500.1.1	*SLmTERF18*	368	42,229.61	9.73	101.11	−0.042	28.78
Solyc11g022600.2.1	*SLmTERF19*	202	24,224.60	9.69	86.39	−0.253	58.63
Solyc02g093950.3.1	*SLmTERF20*	1222	134,350.96	7.99	38.02	0.103	101.82
Solyc02g082010.2.1	*SLmTERF21*	567	64,440.59	9.55	97.09	0.053	46.81
Solyc02g067960.2.1	*SLmTERF22*	435	50,317.11	9.58	97.52	−0.116	41.46
Solyc04g072520.2.1	*SLmTERF23*	346	39,620.67	9.60	96.88	−0.178	30.62
Solyc04g072550.2.1	*SLmTERF24*	514	59,546.28	9.55	104.59	0.076	32.91
Solyc05g007840.3.1	*SLmTERF25*	340	38,787.68	8.86	110.59	0.106	43.82
Solyc09g097920.2.1	*SLmTERF26*	493	56,722.41	8.87	100.75	−0.100	41.31
Solyc11g044360.2.1	*SLmTERF27*	345	39,458.31	9.25	98.23	−0.170	38.39
Solyc11g017050.2.1	*SLmTERF28*	781	87,309.93	5.67	98.00	−0.100	39.10

**Table 2:** Secondary structure of the *mTERF* gene family in tomato.

ID	Alpha Helix	Extended Strand	*Random Coil*
SLmTERF11	49.23%	15.82%	34.95%
SLmTERF13	52.09%	14.74%	33.17%
SLmTERF15	50.39%	11.63%	37.98%
SLmTERF16	40.00%	19.60%	40.40%
SLmTERF17	45.87%	14.81%	39.32%
SLmTERF18	44.29%	20.65%	35.05%
SLmTERF19	45.05%	12.38%	42.57%
SLmTERF21	51.32%	14.99%	33.69%
SLmTERF23	41.91%	17.34%	40.75%
SLmTERF24	51.36%	15.95%	32.68%
SLmTERF9	35.35%	14.88%	49.77%
SLmTERF22	59.54%	6.90%	33.56%
SLmTERF26	46.04%	14.60%	39.35%
SLmTERF4	41.64%	15.85%	42.51%
SLmTERF5	44.60%	12.54%	42.86%
SLmTERF7	47.79%	13.10%	39.12%
SLmTERF8	43.00%	14.40%	42.61%
SLmTERF1	30.71%	19.92%	49.38%
SLmTERF2	38.89%	15.36%	45.75%
SLmTERF3	48.43%	12.37%	49.38%
SLmTERF6	46.95%	14.47%	38.58%
SLmTERF10	48.15%	7.96%	43.89%
SLmTERF12	39.43%	15.14%	45.43%
SLmTERF14	42.86%	13.61%	43.54%
SLmTERF20	49.18%	13.83%	36.99%
SLmTERF25	37.94%	18.82%	43.24%
SLmTERF27	49.86%	15.65%	34.49%
SLmTERF28	38.80%	13.83%	47.38%

## Data Availability

All data are displayed in the manuscript and Supplementary Files.

## Supplementary Materials

The following supporting information can be downloaded at: https://www.mdpi.com/article/10.3390/plants12152862/s1. Table S1. Subcellular localization of 28 SLmTERF proteins; Table S2. Primers for fluorescent quantitative PCR of tomato mTERF gene family and *SLmTERF13* gene silencing; Table S3. YFP-*SLmTERF13* gene PCR primers; Figure S1. Prediction of the *SLmTERF3* signal peptide.
