# Supplementary material for: Analysis of the Tomato mTERF Gene Family and Study of the Stress Resistance Function of SLmTERF-13
Source: Plants (Basel). 2023 Aug 3;12(15):2862. doi: 10.3390/plants12152862 (PMC10421145; doi:10.3390/plants12152862)
Supplement: Supplementary file 1 [file plants-12-02862-s001.zip › plants-2520858-supplementary.pdf]

Table S1. Subcellular localization of 28 SLmTERF proteins.

GENE ID	Prediction	Other location	Signal peptide	Mitochondrial transfer peptide	Chloroplast transfer peptide	Thylakoid luminal transfer peptide
SLmTERF1	Chloroplast	0.197675	0.000006	0.003058	0.75657	0.042691
SLmTERF2	Chloroplast	0.143444	0.001947	0.000209	0.801083	0.053318
SLmTERF3	Mitochondria	0.235126	0.048539	0.390118	0.280796	0.045422
SLmTERF4	Other	0.514763	0.000858	0.245457	0.207987	0.030935
SLmTERF5	Other	0.954047	0.005625	0.033411	0.002603	0.004314
SLmTERF6	Mitochondria	0.266384	0.00003	0.731816	0.001021	0.000749
SLmTERF7	Mitochondria	0.187418	0.000375	0.811325	0.000842	0.00004
SLmTERF8	Other	0.721259	0.034931	0.009497	0.225623	0.00869
SLmTERF9	Chloroplast	0.186059	0.046106	0.029587	0.733313	0.004935
SLmTERF10	Mitochondria	0.103599	0.000001	0.895282	0.000931	0.000187
SLmTERF11	Mitochondria	0.030012	0.00009	0.957794	0.01121	0.000895
SLmTERF12	Mitochondria	0.372148	0.00025	0.626318	0.000721	0.000564
SLmTERF13	Other	0.798832	0.000069	0.000081	0.198547	0.002471
SLmTERF14	Mitochondria	0.188703	0.00082	0.733854	0.033438	0.043185
SLmTERF15	Mitochondria	0.064389	0.008412	0.919507	0.006559	0.001133
SLmTERF16	Mitochondria	0.019599	0.000091	0.980087	0.000215	0.000008
SLmTERF17	Mitochondria	0.041711	0.002902	0.951918	0.003386	0.000083
SLmTERF18	Mitochondria	0.066356	0.014027	0.917848	0.00175	0.00002
SLmTERF19	Other	0.974325	0.000052	0.018681	0.004573	0.002368
SLmTERF20	Other	0.997495	0.001683	0.000618	0.000133	0.00007
SLmTERF21	Other	0.95512	0.044245	0.000609	0.000023	0.000003
SLmTERF22	Other	0.418844	0.006053	0.3503	0.223404	0.001399
SLmTERF23	Other	0.996778	0.000624	0.002543	0.000047	0.000008
SLmTERF24	Other	0.975382	0.010421	0.001377	0.000008	0.012812
SLmTERF25	Chloroplast	0.045233	0.000248	0.000945	0.945229	0.008345
SLmTERF26	Other	0.996669	0.00041	0.002142	0.000466	0.000313
SLmTERF27	Other	0.999938	0.000057	0.000002	0.000002	0.000001
SLmTERF28	Other	0.998608	0.000506	0.000878	0.000007	0.000001

Table S2. Primers for fluorescent quantitative PCR of tomato mTERF gene family and *SLmTERF13* gene silencing.

Primer name	Sequence (5′-3′)	Primer name	Sequence (5′-3′)
SLmTERF1-F	CTAGGTTTGGCCAGCCGTTA	SLmTERF1-R	CTTCAGGCGTTGTCGATCCT
SLmTERF2-F	ACCATCGTCATCAGCATCATGT	SLmTERF2-R	GTGTAAAGACGCGGTGTGAAG
SLmTERF4-F	AGAACCTGGATGCCGAACTT	SLmTERF4-R	GGGAAAAAGGAACACGTACAC
SLmTERF5-F	AACGCTACTTCCCAACCCAA	SLmTERF5-R	TGTAAAATCGCTTGGGGCCT
SLmTERF6-F	TGGTTTCGGAGTTGCCTGAT	SLmTERF6-R	AATTGGCACGACACCAGAGT
SLmTERF8-F	TACTGCTCAAATGCAACGCC	SLmTERF8-R	ATAGCATCACCCCGGAATCG
SLmTERF10-F	AACATGTTCCATCGACCCCG	SLmTERF10-R	CTCTCGTAGCAAAGCAGCAC
SLmTERF11-F	GATCTCCATCCAACTGCGCT	SLmTERF11-R	CGACCGGATATGGTAAGGGC
SLmTERF13-F	GGGTGAACGAAATGCTCGTC	SLmTERF13-R	TGTTCCGAGTTGAGGTTGAGT
SLmTERF14-F	TCTCACCGTGAAAATCTCCGT	SLmTERF14-R	AGCCTGGGAGAAGCCTTTAG
SLmTERF17-F	GCAGCTGTGCCTTACCTTCT	SLmTERF17-R	GTGTGAACGGACGCATTTGT
SLmTERF18-F	CCTTGAAGGTGTTGCCCAGT	SLmTERF18-R	TCTCTGTGAAATCGGTCGGC
SLmTERF19-F	GCGGAGTTCCTGGCATCCTA	SLmTERF19-R	CATCCCTGAAGTGAGAGGCA
SLmTERF20-F	TGCTGCTATTGGTTTTGCCG	SLmTERF20-R	CAGCCGAGAGACAAGGTGTT
SLmTERF21-F	CACTGCACCATTTACTGTCGC	SLmTERF21-R	TGTTCTCTAGTTTTGCCCTGAA
SLmTERF22-F	AGCTGCTGTCTTGCCTTCTC	SLmTERF22-R	TGTCATCGTCGCTACAACCC
SLmTERF23-F	AGTTTTGCCCCGGTTGGAAT	SLmTERF23-R	CATCAGGCAGCTTATGGGCA
SLmTERF27-F	AGAGGCAGCTTATCCGTTCG	SLmTERF27-R	GCCAGAACTGTGGCTGTTTG
SLmTERF28-F	GACAAAAGCGAATCCAGCCC	SLmTERF28-R	GCAGAACGAATCTGCTGTCC
Actin-7-F	ATTGGTGCTGAGAGGTTCCG	Actin-7-R	CGGGAAACAGACAGGACACT

SLmTERF13-F （gene silencing）	GTGAGTAAGGTTACCG AATTCTTTCCTTATGGA TTTCATACGTTA	SLmTERF13-R (gene silencing)	CGTGAGCTCGGTACC GGATCCCTCAAGCTT CTTTT CGTCCAA

Table S3. YFP-*SLmTERF13* gene PCR primers.

Primer name	Sequence	Primer length	Product length
YFP-*SLmTERF13*-F	ACGGGGGACTCTAGAGGATCCATGCTCG TCA GATTCAGAGG	20	1107
YFP-*SLmTERF13*-R	GCCCTTGCTCACCATGGTACCAGGAGCC AGA GTTTTCTGG	19	

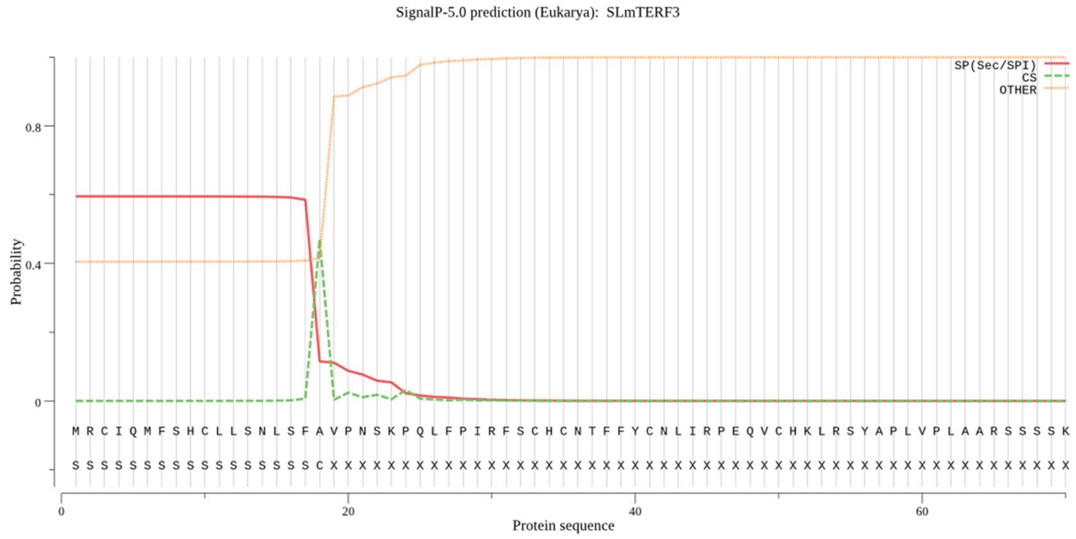

Figure S1. Prediction of the *SLmTERF3* signal peptide.
